# The Structural Variation Is Associated with the Embryonic Lethality of a Novel Red Egg Mutant Fuyin-*lre* of Silkworm, *Bombyx mori*

**DOI:** 10.1371/journal.pone.0128211

**Published:** 2015-06-01

**Authors:** Anli Chen, Pengfei Liao, Qiongyan Li, Qiaoling Zhao, Weike Yang, Shuifen Zhu, Fang Wu, Rongfan He, Zhanpeng Dong, Ping Huang

**Affiliations:** 1 The Sericultural and Apicultural Research Institute, Yunnan Academy of Agricultural Sciences, Mengzi Yunnan, China; 2 Sericultural Research Institute, Chinese Academy of Agricultural Sciences, Zhenjiang Jiangsu, China; Institute of Plant Physiology and Ecology, CHINA

## Abstract

*Bombyx mori* presents several types of egg color mutations, all of which have been extensively discussed in sericulture. While the red egg mutation has been previously observed, lethal red-egg mutants have not been reported. In the present work, the red egg mutant Fuyin-*lre* (Fuyin-lethal red egg) was discovered from the Fuyin germplasm resource of *B*. *mori*. This mutant features red-colored eggs and embryonic lethality. Genetic analysis showed that Fuyin-*lre* follows recessive inheritance, with the red egg gene *re* governing the egg color, and the embryonic lethality of Fuyin-*lre* may be caused by mutations of other genes closely linked to *re*. Digital gene expression (DGE) was employed to compare the transcription profiles of Fuyin and Fuyin-*lre* eggs after 24 and 48 h of incubation. A total of 48 differentially expressed genes followed the same expression patterns in both groups at both time points (FDR < 0.01 and *log* 2 Ratio ≥ 1). Further analyses indicated that 8 out of the 48 genes (including *re*) were closely linked to *re*. These 8 genes were highly expressed in wild-type Fuyin and the red egg mutant *re* but showed nearly absent expression in Fuyin-*lre*. Sequencing of the *re* gene confirmed that the *re* gene itself does not induce embryonic lethality, and structure analysis showed that the structural variation of the region where the 8 genes were located may be associated with the embryonic lethality of Fuyin-*lre*. The present work provides a good foundation for future studies on the mechanism of embryonic lethality and embryonic development in Fuyin-*lre*.

## Introduction

*Bombyx mori* (domesticated silkworm) of the order Lepidoptera is an agriculturally and economically important insect. Silkworms have been used as experimental insects for studies in genetics, modern molecular biology, and genetic engineering. The genetic mutations of *B*. *mori* are an important aspect of sericulture. The Silkworm Genome Database of Southwest University contains over 90% of the world's silkworm mutant resources, and considerable progress has been made in the linkage analysis and development of marker systems for gene positioning [1–2]. Since the Silkworm Genome Research Program was completed [3], various mutated gene resources of *B*. *mori* have become important materials for studies on gene functions.

Egg color is an important trait in morphological and genetic studies of *B*. *mori*. Egg color mutations may occur in the egg shell or serous membrane. The color of the serous membrane is formed after fertilization. There are many types of color mutants, including white, brown, red, purple, orange, rust-colored eggs [4–6], and so on. The important red egg mutants of *B*. *mori* may be classified as red (*re*) [7–8], fourth brown (*b-4*), orange (*ci*) [9], and rusty (*re/reci/ci*) egg mutants [10]. Osanai-Futahashi et al. [8] performed positional cloning of the red egg gene *re* in 2012. Unlike the discovery of mutants, the identification of mutated genes in egg mutants has lagged behind. In particular, the traditional identification methods are time- and labor-consuming, and research progress is relatively slow.

Completion of the Silkworm Genome Sequencing Project has enabled studies of mutant genes via molecular markers, genome resequencing, and digital gene expression (DGE) analysis. Researchers from China have completed the draft genome sequence [3] and fine genome map [11] of *B*. *mori* as well as the map of genome variation in 40 domestic and wild-type silkworms [12]. The fine genome map of *B*. *mori* can reach up to 99.6% coverage. Moreover, 76.7% of the genomic fragments and 82.2% of the genes have been localized to their respective *B*. *mori* chromosomes. These advances have greatly accelerated the localization and identification of the mutated genes.

Fuyin-*lre* is a novel red egg mutant that was first discovered in the *B*. *mori* germplasm resources of Fuyin. This mutant differs from previously reported red egg mutants because Fuyin-*lre* presents embryonic lethality. Fuyin-*lre* eggs stop developing approximately 48 h after incubation, thereby exhibiting recessive lethal mutation. Thus, significant impacts may be achieved with the maintenance of this variety. In the present study, we analyzed the embryonic development and mode of inheritance of Fuyin-*lre* and compared differentially expressed genes (DEGs) in wild Fuyin and mutant Fuyin-*lre* embryos at different developmental stages. The structural variation of the red egg gene *re* (BGIBMGA003497-1) was found to be the direct reason for the red egg trait in Fuyin-*lre*. The embryonic lethality of Fuyin-*lre* may be related to silencing of all 8 genes closely linked to red egg gene *re*. The present work provides a good reference for future research on the mechanisms of embryonic lethality and development.

## Materials and Methods

### Silkworm strains

**Fig 1:**
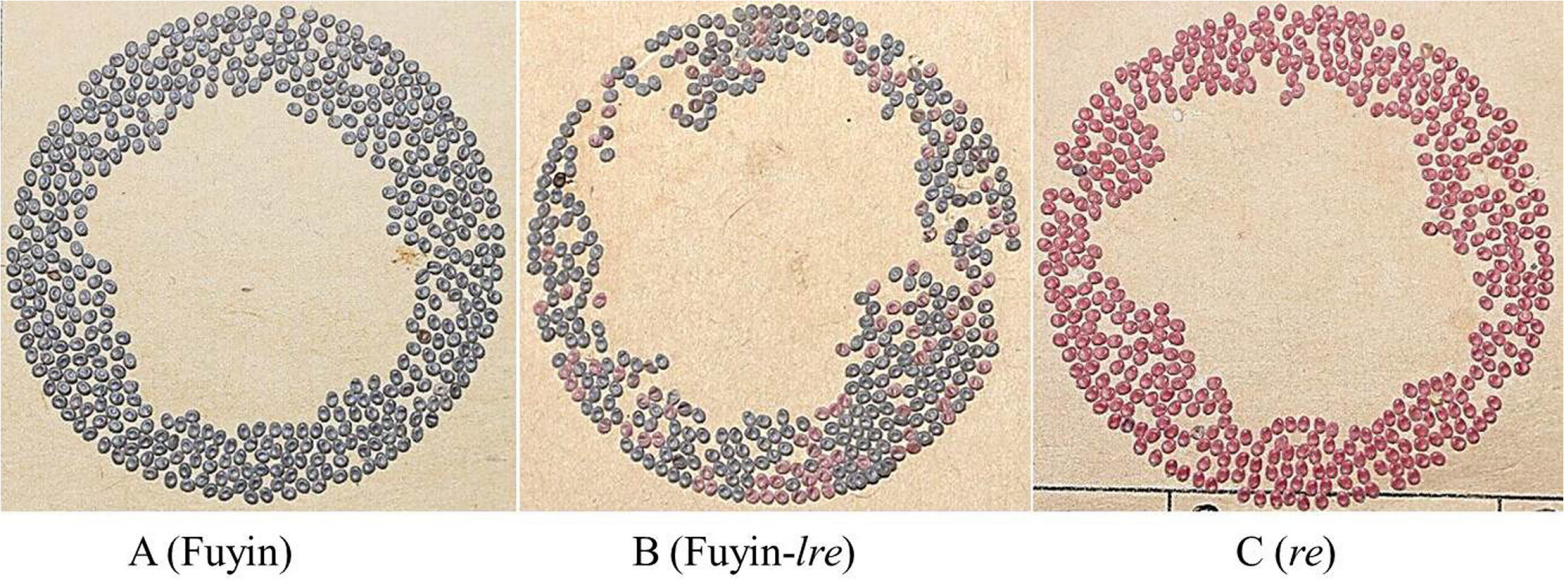
Phenotypes of eggs from three varieties of *Bombyx mori*. All eggs within one ring were laid by the same female moth. (A) Eggs laid by wild Fuyin hatched normally after incubation. (B) Fuyin-*lre* naturally mutated from wild Fuyin. Normal eggs in the ring hatched normally after incubation, but red eggs did not. (C) Red eggs laid by red egg mutant *re*, and the red color of eggs obtained as a result of mutation of the red egg gene *re*. All of the red eggs in the ring hatched normally.

Wild-type Fuyin (Fig 1A) and its mutant Fuyin-*lre* with embryonic lethality (Fig 1B) were preserved by the Sericultural and Apicultural Research Institute, Yunnan Academy of Agricultural Sciences. The red egg mutant *re* (Fig 1C) was obtained from Southwest University. All *B*. *mori* individuals were fed with fresh mulberry leaves at a constant temperature of 25 ± 0.5°C and constant humidity of 75%‒80%.

### Genetic analysis of Fuyin-*lre*

Normal eggs of the Fuyin-*lre* ring were conventionally reared until they reached adulthood. Inbreeding of Fuyin-*lre* and its cross breeding with the red egg mutant *re* were performed. The segregation of normal and red eggs in F_1_ offspring from inbreeding and cross breeding was investigated. Inbreeding was performed, in turn, on the offspring obtained from cross breeding, and subsequent segregation of F_2_ was determined.

### Anatomy of the embryos, and extracting total RNA extraction and genomic DNA from eggs

Eggs laid by Fuyin, Fuyin-*lre*, and *re* were separately treated and incubated [13]. Every 24 h after incubation, the eggs were treated with 20% NaOH at 100°C for 3-5s, and then, eggs were blew with a dropper to separate the embryo from eggshell in clean water. The embryonic morphology of these eggs was observed under a microscope to determine the developmental stages of the embryo.

Every 24 h after incubation, 0.5 g of normal eggs laid by Fuyin as well as red eggs laid by Fuyin-*lre* and *re* was collected for total RNA extraction using the RNAiso Plus (TaKaRa) kit. The extracted RNA was digested by DNase I (TaKaRa) to remove DNA residues. The concentration and purity of total RNA was determined using a BioMate 3S UV-visible spectrophotometer (Thermo Fisher Scientific), and the quality of total RNA was identified using an Agilent bioanalyzer (Agilent Technologies 2100).The qualified RNA was used in DGE and qRT-PCR.

Normal eggs laid by Fuyin and the red eggs laid by Fuyin-*lre* and *re* were selected to extract their genomic DNA according to the literature [14]. Each qualified DNA sample was confirmed by the BioMate 3S UV-visible spectrophotometer before dilution to 100 ng/μL and storage at—20°C.

### Illumina sequencing

Total extracted RNA from normal eggs from Fuyin and red eggs from Fuyin-*lre* at different developmental stages were selected according to the anatomical results of Fuyin-*lre* embryos. mRNA enrichment was performed using magnetic beads with Oligo (dT), and the RNA concentration of the samples was between 416 and 680 ng/μl. Relevant reagents were added to the mRNA obtained for fragmentation at high temperature. The fragmented mRNA was then used as a template to synthesize cDNA. Magnetic bead purification, end repair, addition of base A to the 3ʹ-end, and addition of adaptors were performed prior to PCR amplification. The library was subsequently constructed. Library quality and production were determined by an Agilent 2100 Bioanalyzer and an ABI StepOnePlus Real-Time PCR System, respectively. Qualified mRNA was sequenced by IlluminaHiSeq 2000. Raw data presented in this publication have been deposited in the NCBI Sequence Read Archive (http://www.ncbi.nlm.nih.gov/sra/) and are accessible through SRA accession number: SRP053552.

### Data analysis

Original images produced by the sequencer were converted into raw data by Base Calling software. Low-quality and adaptor sequences were removed to obtain clean reads for subsequent analysis. Reference genome sequences of *B*. *mori* were downloaded from SilkDB (http://silkworm.swu.edu.cn/silkdb/), and clean reads were aligned with reference genome sequences using Tophat software [15]. Gene expression was calculated by the RPKM (reads per kb per million reads) algorithm [16]. The expression levels of genes in different samples were compared to screen differentially expressed genes (DEGs). The sequencing-based method reported by Audic et al. [17] was adopted to analyze DEGs at two time points. The *P*-value of the difference test was corrected by multiple hypothesis testing, and the threshold of *P*-value was determined by controlling the false discovery rate (FDR). FDR is a technique used to determine the threshold of *P*-value during multiple comparison and analysis. The smaller the FDR, the larger the fold change and the more significant the expression difference. Genes with FDR ≤ 0.01 [18] and *log* 2 ratios ≥ 1 were considered DEGs. The DEG sequences were BLAST-searched, mapped, annotated, and analyzed using Kyoto Encyclopedia of Genes and Genomes (KEGG) metabolism pathways and Blast2GO software (version 2.7.2) according to the Blast2GO Tutorial [19].

### qRT-PCR analysis

The criteria for DEGs screening were based on the results of DGE analysis. qRT-PCR detection was performed on screened genes to verify the reliability of DGE analysis. Based on the embryonic anatomy of Fuyin-*lre*, 2 μg of the total RNA of normal eggs by Fuyin, red eggs by Fuyin-*lre*, and red eggs by *re* were used to synthesize first-strand cDNA with a PrimeScript reverse transcriptase kit (TaKaRa) in a 20 μL reaction system. The cDNA products were diluted by fivefold with ddH_2_O. Each 20 μL qRT-PCR reaction system consisted of 9 μL of SYBR Premix Ex Tag (Roche, 2×), 0.8 μL of the specific primers, 1 μL of cDNA template, and 9.2 μL of ddH_2_O; here, three replicates were produced. qRT-PCR was performed in a StepOnePlus real-time PCR system (Applied Biosystems) using default parameters. The housekeeping *B*. *mori* actin 3 gene (GenBank ID: NM_001126254) [20] was used as a reference to eliminate bias among samples, and qRT-PCR results were converted and calculated via the 2^-ΔΔ*Ct*^ method [21]. The primers used for qRT-PCR are shown in S1 Table.

### Identification of mutation site

To investigate the mutation site, several pairs of primers were designed for PCR amplification among Fuyin, Fuyin-*lre*, and *re* according to the results of DGE and qRT-PCR. The reference genomic sequence was obtained by blasting against the silkworm genome database (http://www.silkdb.org/silkdb/). *Bmactin* 3 gene was used as a reference to eliminate difference among samples. PCR reactions were carried out under the following conditions: 1 cycle of 94°C for 5 min, 31 cycles of 94°C for 30 s, 55–58°C for 30 s and 72°C for 1 min, with a final extension step of 5 min at 72°C. About 6 μL of amplified DNA products were then separated on 1.0% agarose gels, and visualized with UV transilluminator. The primers used for PCR amplification are shown in S2 Table.

## Results and Discussion

### Inheritance rule of Fuyin-*lre*

Single batch rearing and inbreeding was performed on normal eggs of Fuyin-*lre* to determine the inheritance rule of the mutants. For each generation of inbreeding, normal and mutant egg batches were present. After nine generations of inbreeding, four batches with the red egg mutation were randomly selected to count the number of normal and red eggs. Results showed that the ratio of normal eggs to red eggs was 3:1. The hatching rate of normal eggs exceeded 90%, whereas that of the red eggs was 0% (Table 1). Results of inbreeding showed that Fuyin-*lre* is controlled by a recessive gene. Red egg individuals in the batch showed the genotype *lre*/*lre*, whereas normal individuals showed two genotypes (+/+ and +/*lre*). The +/+:+/*lre*:*lre*/*lre* ratio was 1:2:1.

**Table 1:** Segregation and hatchability of normal and red eggs laid by Fuyin-*lre*.

Batch number	Number of normal eggs / hatching rate (%)	Number of red eggs / hatching rate (%)	Segregation ratio	$\chi_{\text{c}}^{2}$
1	449/96.32	138/0	3:1	0.62
2	410/91.75	121/0	3:1	1.27
3	359/93.34	110/0	3:1	0.52
4	331/95.56	123/0	3:1	0.95
$\bar{x}$	387.25/94.24	123/0	3:1	0.17

$\chi_{\text{c}}^{2}$ is used for detecting the fitness of genetic segregation ratio, when $\chi_{\text{c}}^{2}$ < $\chi_{0.05,1}^{2}$ = 3.84, the offspring segregation ratio of inbred consistent with phenotypic segregation ratio of a pair alleles.

To determine whether or not the *re* gene controls the red egg trait of Fuyin-*lre*, normal individuals of Fuyin-*lre* (+/+ or +/*lre*) were hybridized with the red egg mutant *re*. The F_1_ generation of the reciprocal cross showed normal and red egg batches. All of the eggs in the normal egg batch were normal. The ratio of normal egg to red egg numbers in the red egg batch was 1:1, and the eggs of both colors could be normally hatched. The normal and red eggs of the F_1_ generation in the red egg batch were subsequently collected and separately reared. And then, three mating patterns were observed: normal × normal (N×N), normal × red (N×R), and red × red (R×R). Results indicated that red eggs appear in all batches of N×N, with a normal egg to red egg number ratio of 3:1. Red eggs also appeared in all batches of N×R with a segregation ratio of 1:1. All eggs in R×R batches were red. All eggs of the three different phenotypes were incubated by pickling, and all of the eggs in the batches showed segregation ratios of 3:1 or 1:1 could be hatched normally. The hatching rate of red eggs in the all-red-egg batches was 72%‒74%. The ratio of the number of hatched eggs to that of un-hatched eggs satisfied the law of segregation (Table 2). The results described above indicate that the gene controlling the red egg trait of Fuyin-*lre* is *re*. As all of the red eggs in batches of Fuyin-*lre* could not be hatched, the genes related to embryonic lethality are probably the genes very closely linked to *re*.

**Table 2:** Segregation of spring from the reciprocal cross of *lre* and *re*.

				Mutant batches			
Cross combinations	Generation	Number of batches of normal eggs	Number of batches of mutant eggs	Number of normal eggs (average of 3 moths) / hatching rate (%)	Number of red eggs (average of 3 moths) / hatching rate (%)	Segregation ratio	$\chi_{\text{c}}^{2}$
*lre* ×*re*	F_1_	21	35	249/97.18	255/94.88	1:1	0.05
*re*×*lre*	F_1_	24	30	212/93.03	229/96.46	1:1	0.58
*lre*×*re* (N×N)	F_2_	0	52	470/91.27	141/96.34	3:1	1.10
*re*×*lre* (N×N)	F_2_	0	53	421/95.55	153/93.31	3:1	0.75
*lre*×*re* (N×R)	F_2_	0	49	301/95.30	322/97.93	1:1	0.64
*re*×*lre* (N×R)	F_2_	0	56	297/93.24	272/92.70	1:1	1.01
*lre*×*re* (R×R)	F_2_	0	54	0/—	592/73.48	-	-
*re*×*lre* (R×R)	F_2_	0	50	0/—	548/72.44	-	-

$\chi_{\text{c}}^{2}$ is used for detecting the fitness of genetic segregation ratio, when $\chi_{\text{c}}^{2}$ < $\chi_{0.05,1}^{2}$ = 3.84, the offspring segregation ratio of various mating form consistent with phenotypic segregation ratio of a pair alleles.

### Embryonic development of Fuyin-*lre*

**Fig 2:**
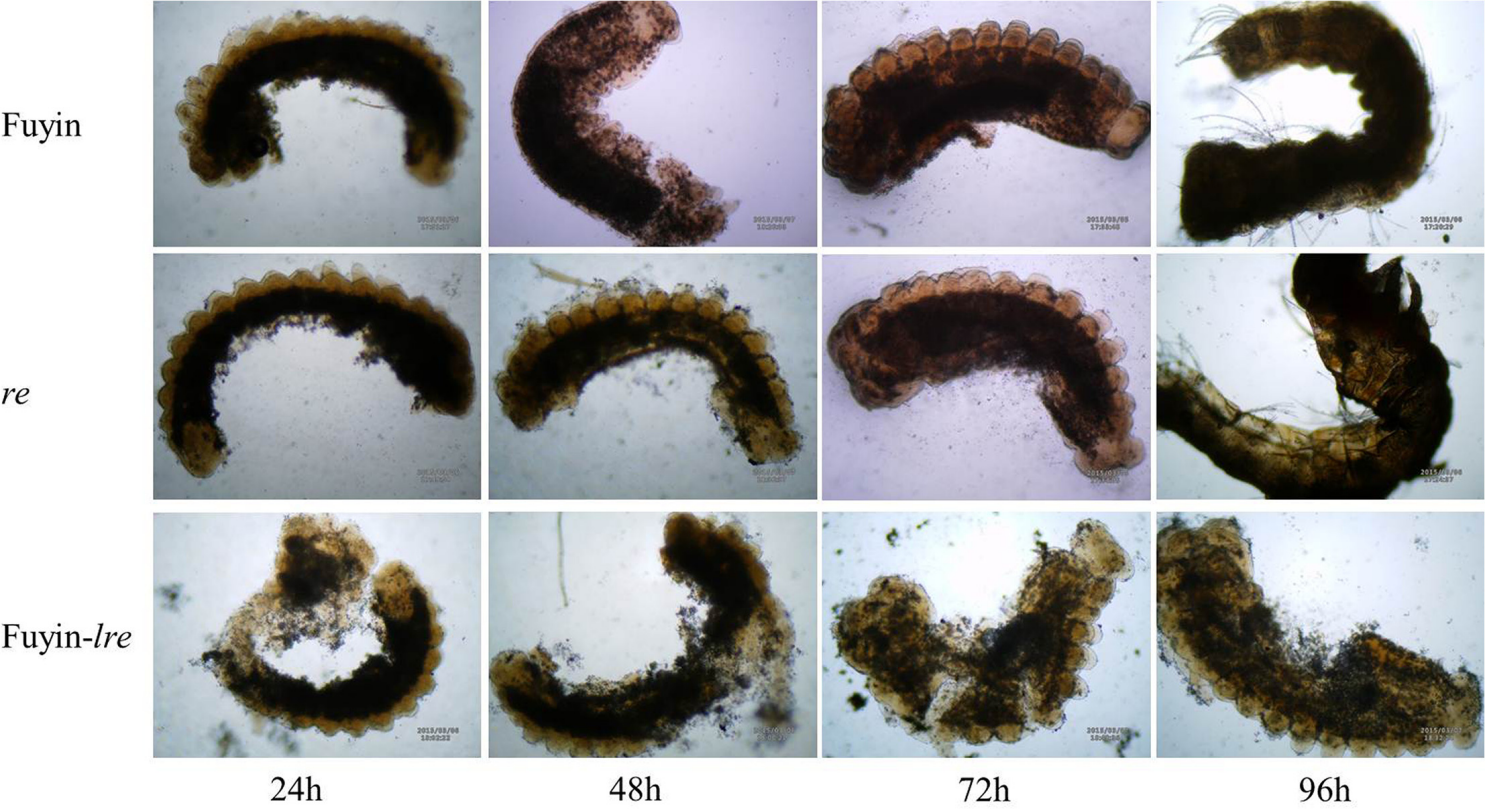
Embryos morphology of Fuyin, *re*, and Fuyin-*lre* at different developmental stages (10×40). Fuyin and *re* embryos could develop normally. However, Fuyin-*lre* embryos stop developing after approximately 48 h of incubation, and it was difficult to obtain intact embryos.

To determine the last stage of development of Fuyin-*lre* embryos, red eggs were dissected with the method mentioned in the Materials and Methods. The Fuyin-*lre* embryos strongly adhered to the egg shell, thereby rendering dissection difficult. Intact embryos were difficult to obtain, and a large amount of adherent substances were present; thus, only a rough contour of the embryo was visualized (Fig 2). The embryonic body of the aborted embryos was fairly long and showed an enlarged head and tail folds. Depressions on the head fold, abdomen, and tail were only indistinctly visualized. No longitudinal groove was observed in the first and the second segments. This morphology resembles that of the normal embryo at approximately 48 h of incubation. Therefore, we hypothesized that Fuyin-*lre* embryos stop developing after approximately 48 h of incubation.

### DGE profiles

The embryonic anatomy of Fuyin-*lre* showed that the embryos stop developing after approximately 48 h of incubation. Therefore, the eggs of wild-type Fuyin and its mutant Fuyin-*lre* obtained after 24 and 48 h of incubation were selected as materials to construct four DGE tag libraries. High-throughput sequencing was performed with HiSeq 2000 (Illumina) to analyze differences in transcriptional levels in Fuyin and Fuyin-*lre* embryos at 24 and 48 h.

Sequencing produced 11.7‒12.3 million raw reads for each sample (S1 Fig). After removal of adaptor-containing reads, reads with an *N* proportion larger than 10%, and low-quality reads (the number of bases with Q ≤ 5 accounted for over 50% of raw reads), the number of clean reads accounted for over 99.5% of the raw reads of each example; these values far exceed the lowest standards of sequencing (clean reads/raw reads ≥ 90%, N ≤ 2%, adaptor ≤ 2%). Sequencing saturation analysis can be used to determine whether or not the reads meet the required standard. In this study, sequencing results showed that the number of detected genes increases very slowly when the number of reads is approximately 3.0 M (S2 Fig). These results confirm the high reliability of the sequencing results.

### DEGs analysis

The DEGs of Fuyin and Fuyin-*lre* obtained after 24 and 48 h were detected using the RPKM algorithm [16] and by referring to the detection method of Audic et al. [17]. Here, FDR ≤ 0.001 and *log* 2 ratios ≥ 1 were selected for DEG screening. The results identified 82 DEGs in Fuyin and Fuyin-*lre* at 24 h; of these genes, 30 genes were upregulated and 52 genes were downregulated. At 48 h, 237 DEGs in Fuyin and Fuyin-*lre* were detected, among which 99 genes were upregulated and 138 genes were downregulated. DEGs with the same expression trends at the two time points (simultaneously upregulated or downregulated) were analyzed to confirm screening accuracy. A final total of 48 DEGs were selected for subsequent analysis; here, 17 genes were upregulated and 31 genes, including the red egg gene *re* (BGIBMGA003497-1), were downregulated (S3 Table).

**Fig 3:**
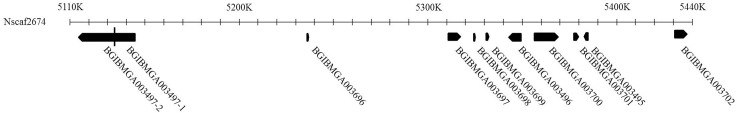
Position of DEGs linked to *re* on the chromosome. *BGIBMGA003497* actually consisted of two different genes, and *BGIBMGA003497-1* gene was responsible for the *re* phenotype, it had been proved by Mizuko Osanai-Futahashi [8]. *BGIBMGA003497-2* was a function unknown gene, and not included in DEGs which linked to *re*.

Genetic analysis indicated that genes related to the embryonic lethality of Fuyin-*lre* may be genes closely linked to *re*. Thus, the 48 DEGs were aligned with the reference genome (http://www.silkdb.org/silkdb/), and 8 genes (including *re*, Table 3), namely, BGIBMGA003497-1 (*re*), BGIBMGA003697, BGIBMGA003698, BGIBMGA003699, BGIBMGA003496, BGIBMGA003700, BGIBMGA003701, and BGIBMGA003495, which were closely linked to *re* were found. BGIBMGA003495-TA is the gene farthest from *re*, with a distance of approximately 250 kb (Fig 3). Alignment with the reference genome revealed 9 genes in this region. Besides the 8 DEGs previously mentioned, another gene BGIBMGA003696 was relevant, although this gene was not included in the DGE results. According to DGE analysis of two genes (BGIBMGA003497-2 and BGIBMGA003702) flanking this region, no obvious differences in the expression of the two genes in Fuyin and Fuyin-*lre* could be observed.

**Table 3:** DGE and qRT-PCR results of the 9 genes from BGIBMGA003497-1 to BGIBMGA003495.

		Fuyin-*lre* / Fuyin		
Genes	Silkworm Genome Datebase	24h / 48h Fold^a^ 24h / 48h Fold^b^		Up / Down
BGIBMGA003497-1	major facilitator superfamily domain-containing protein 12-like (Bm-*re*) [*Bombyx mori*]	-11.43 /—	0.012 / 0.003	Down
BGIBMGA003696	No item	— /—	— /—	—/—
BGIBMGA003697	PREDICTED: semaphorin-1A-like [*Bombyx mori*]	-8.02 / -7.92	0.07 / 0.003	Down
BGIBMGA003698	PREDICTED: UPF0585 protein CG18661-like, transcript variant X2 [*Bombyx mori*]	-2.82 / -11.25	0.09 / 0.01	Down
BGIBMGA003699	PREDICTED: LIN1-like protein-like [*Bombyx mori*]	-13.4 / -13.3	0.08 / 0.004	Down
BGIBMGA003496	PREDICTED: GPI ethanolamine phosphate transferase 1-like [*Bombyx mori*]	-11.09 / -11.92	0.084 / 0.012	Down
BGIBMGA003700	PREDICTED: ras GTPase-activating-like protein IQGAP1-like [*Bombyx mori*]	-12.18 / -12.61	1.70E-08 / 0.004	Down
BGIBMGA003701	PREDICTED: metallophosphoesterase 1-like [*Bombyx mori*]	-11.46 / -11.45	0.12 / 0.01	Down
BGIBMGA003495	PREDICTED: protein naked cuticle homolog [*Bombyx mori*]	-7.34 / -13.37	0.09 / 0.004	Down

Fold^0^: Fold change (Fuyin-*lre* / Fuyin) of gene expression in RPKM.

Fold^b^: Fold change (Fuyin-*lre* / Fuyin) of gene expression in qRT-PCR.

### qRT-PCR

To verify the reliability of DGE analysis and compare differences in the expression levels of 9 DEGs (including BGIBMGA003696) between *re* and BGIBMGA003495 in Fuyin, Fuyin-*lre*, and *re* mutants, qRT-PCR was performed. All 8 genes except BGIBMGA003696 were highly expressed in Fuyin and *re* eggs, but the expression of these genes was nearly absent in Fuyin-*lre* (Table 3 and Table 4). However, gene BGIBMGA003696 was absent in all of three traits. This result is consistent with the DGE results, which are considered to be of very high reliability.

**Table 4:** Results of qRT-PCR between Fuyin-*lre* and *re*.

Genes	24h (Fuyin-*lre* / *re*) log2 Ratio qRT-PCR	Up / Down	48h (Fuyin-*lre* / *re*) log2 Ratio qRT-PCR	Up / Down
BGIBMGA003497-1	0.015	down	0.002	Down
BGIBMGA003696	—	—	—	—
BGIBMGA003697	0.29	down	0.06	Down
BGIBMGA003698	0.02	down	0.001	Down
BGIBMGA003699	0.03	down	0.001	Down
BGIBMGA003496	0.05	down	0.01	Down
BGIBMGA003700	0.02	down	0.001	Down
BGIBMGA003701	0.03	down	0.001	Down
BGIBMGA003495	0.33	down	0.52	Down

The results described above show that *re* mutation did not influence the expression of other linked genes in the *re* mutant. Thus, embryonic development proceeded normally except for the fact that the eggs were red-colored. The embryonic lethality of Fuyin-*lre* may be attributed to silencing of all genes closely linked to *re*. The region where the 9 genes are located may have undergone significant structural changes, thereby causing abnormal expression of all the genes in this region. Consequently, the Fuyin-*lre* embryos did not develop normally.

### Structural analysis of *re* and its neighboring sequences

**Fig 4:**
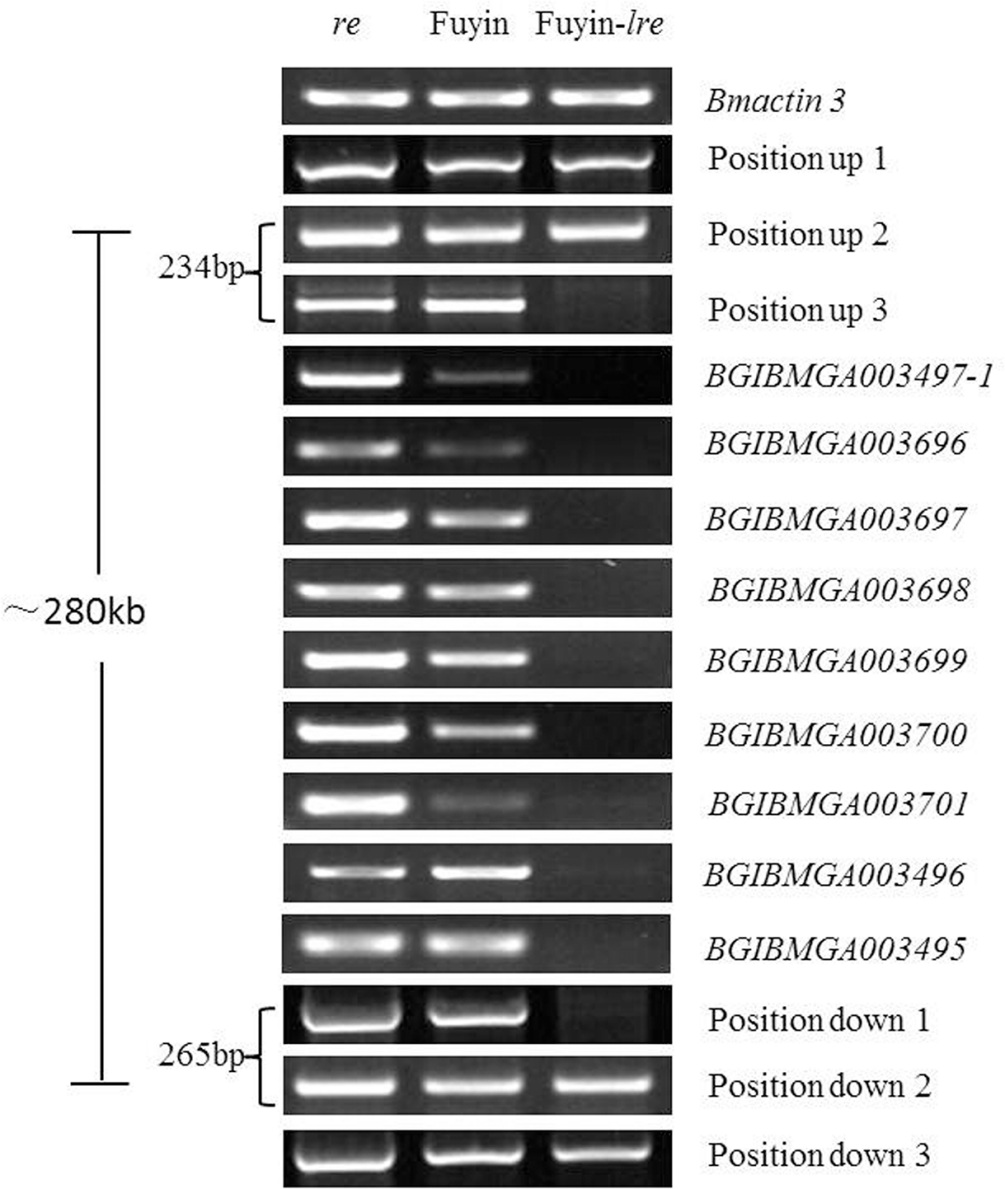
PCR amplification in the region where the 9 genes are located using the Fuyin, Fuyin-*lre*, and *re* genomes as templates. The genotype of Fuyin is +/+.

**Fig 5:**
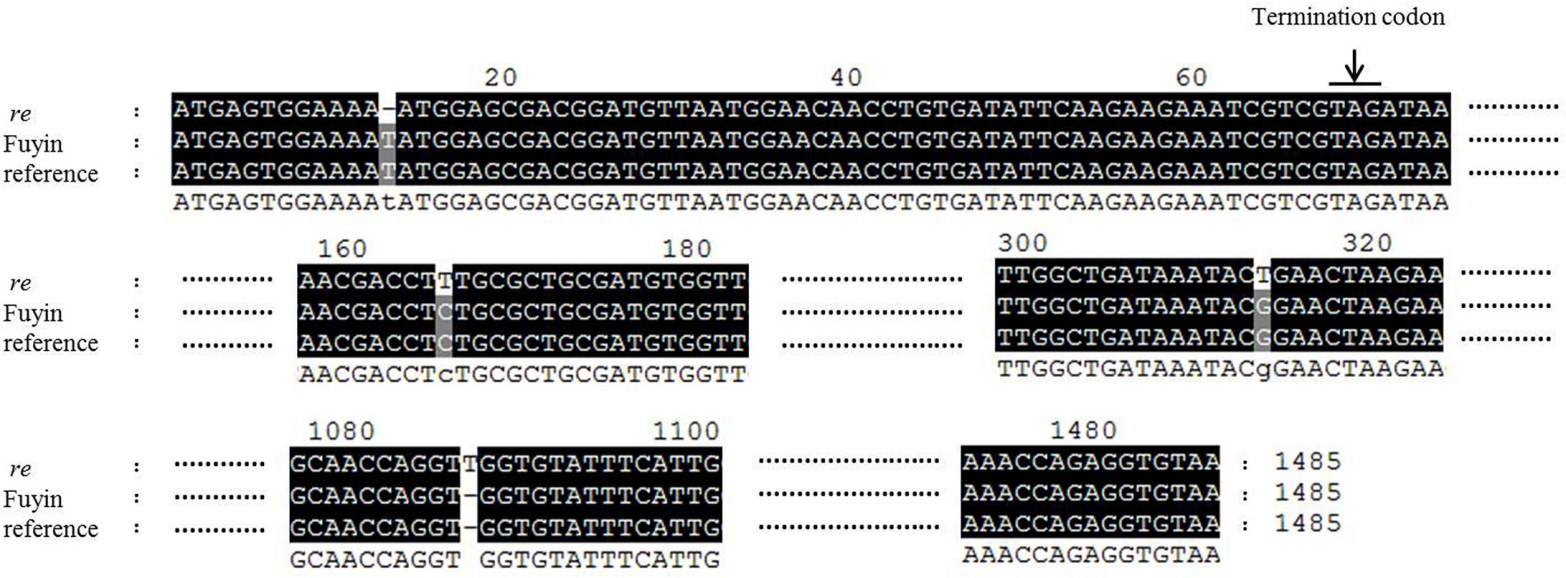
Sequencing of *re* gene in mutant *re* and Fuyin. The genotype of Fuyin is +/+. The reference sequence was obtained by blasting against the silkworm genome database (http://www.silkdb.org/silkdb/).

The hypothesis of structural variation was tested by PCR amplification in the region where the 9 genes described above are located using the Fuyin, Fuyin-*lre*, and *re* genomes as templates. Results indicated that several pairs of primers could produce good amplification effects when the Fuyin and *re* genome was used as a template (Fig 4). However, when the Fuyin-*lre* genome was used as a template, the sequences could not be amplified by PCR. Sequencing results showed that mutation of the *re* gene in the *re* mutant occurs differently from the previously reported mutation in exon 9 of the *re* gene [8]. In this study, a frameshift mutation occurred because of base deletion in exon 1 and the presence of an additional base in exon 8 of the *re* gene (Fig 5). The stop codon that terminates protein translation appeared at the front end of the ORF. Thus, the *re* gene could be transcribed but not translated into the corresponding proteins. This phenomenon could explain the red color of *re* eggs. As demonstrated above, complete functional loss of the *re* gene only produced the red color of the mutant but did not cause embryonic lethality. Given the structural variation of Fuyin-*lre*, the *re* gene could not be transcribed normally (Table 4), which is consistent with the DGE and qRT-PCR results. Therefore, the hypothesis of structural variation in the *re* gene and its neighboring sequences in Fuyin-*lre* mutants was confirmed. The genes between BGIBMGA003497-1 and BGIBMGA003495 (BGIBMGA003696 excluded) were expressed in normal eggs and, except for the *re* gene, have unknown functions (Table 3). Therefore, the specific genes connected with embryonic development are difficult to determine. The specific mode of mutation and key genes related to embryonic development must be identified through further study.

## Conclusion

The Fuyin-*lre* mutant is a newly discovered red egg mutant with embryonic lethality. The mutant produced red-colored eggs and failed to develop normally because of embryonic lethality. Genetic analysis of Fuyin-*lre* revealed that the Fuyin-*lre* mutant is recessive and that the recessive homozygote presents embryonic lethality. DGE analysis and qRT-PCR were performed to compare DEGs in Fuyin and Fuyin-*lre*; 9 genes closely linked to *re* (including BGIBMGA003696) demonstrated structural variation. We confirmed that *re* mutation does not directly cause embryonic lethality. None of the red eggs of Fuyin-*lre* individuals developed normally, which suggests that genes causing the embryonic lethality of Fuyin-*lre* are closely linked to the *re* gene. This result is consistent with the hypothesized structural variation of the 9 genes closely linked to *re* in Fuyin-*lre*. The results of the present work may help guide future investigations on the underlying mechanisms of Fuyin-*lre* mutation and *B*. *mori* embryonic development.

## Supporting Information

S1 Fig Quality assessment of reads. (TIF)

S2 Fig Sequencing saturation analysis. (TIF)

S1 Table Primers used in qRT-PCR. (DOCX)

S2 Table Primers used in positioning of the mutation site. (DOCX)

S3 Table DEGs with the same expression trends at 24h and 48h. (DOCX)
