# Supplementary figures and images for: The Structural Variation Is Associated with the Embryonic Lethality of a Novel Red Egg Mutant Fuyin-lre of Silkworm, Bombyx mori
Source: PLoS One. 2015 Jun 1;10(6):e0128211. doi: 10.1371/journal.pone.0128211 (PMC4452371; doi:10.1371/journal.pone.0128211)

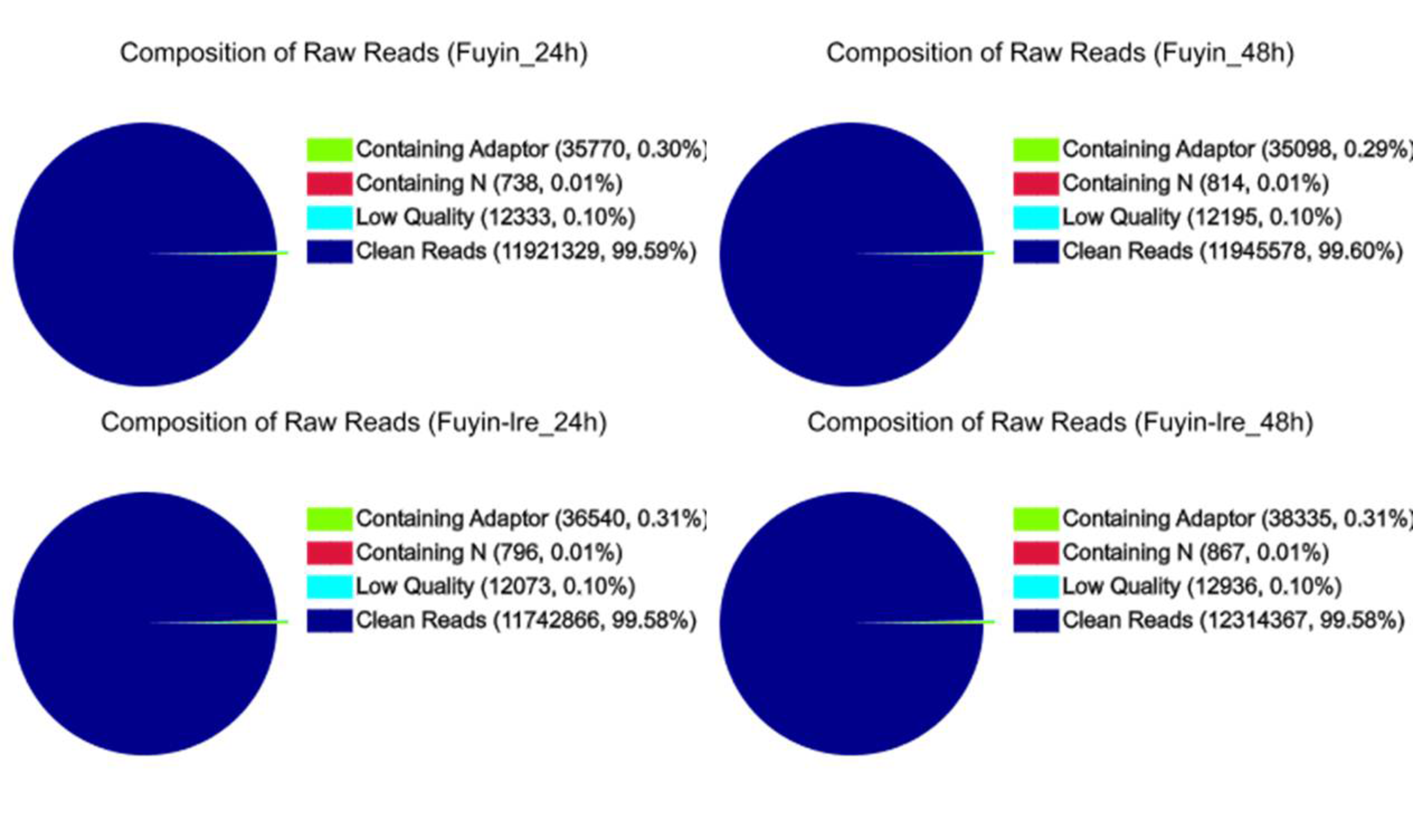

Supplement: S1 Fig — (TIF) [file pone.0128211.s001.tif]

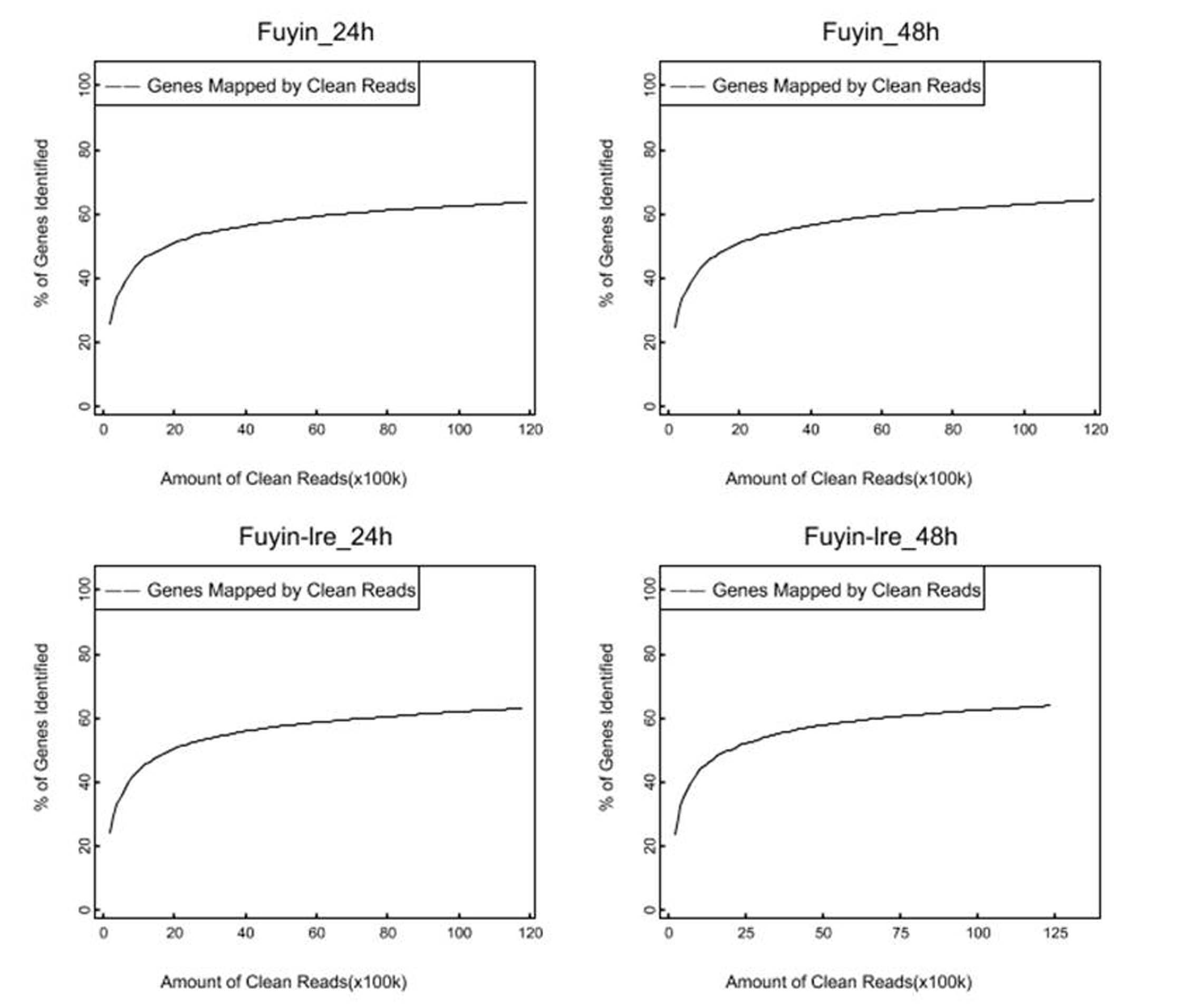

Supplement: S2 Fig — (TIF) [file pone.0128211.s002.tif]
