# Supplementary material for: The Structural Variation Is Associated with the Embryonic Lethality of a Novel Red Egg Mutant Fuyin-lre of Silkworm, Bombyx mori
Source: PLoS One. 2015 Jun 1;10(6):e0128211. doi: 10.1371/journal.pone.0128211 (PMC4452371; doi:10.1371/journal.pone.0128211)
Supplement: S1 Table — (DOCX) [file pone.0128211.s003.docx]

S1 Table Primers used in qRT-PCR

| Primer name | Forward primer | Reverse primer |
| --- | --- | --- |
| BGIBMGA003497-1 | GAAGCTCAATCACGCTCGTCTCCAG | GACAATATGCCAAGTGCGGCAC |
| BGIBMGA003696 | GCATTTACCGTGAAAGCC | AGTATTTACAGGATGGGTTG |
| BGIBMGA003697 | CGTCTTGTCTCGTTACCCT | ACGTTCGCTACCATATTGAC |
| BGIBMGA003698 | AGCAGCAACAAGGAACCAAG | TCTGAAAACCCATAATGAACTA |
| BGIBMGA003699 | AAGAAGATAGTGGTGCTGAA | CAATGGTAATGTCCTTGTGTA |
| BGIBMGA003496 | GCACCAATAATGGCAACG | TGTGCAGCTATTTGTCTACT |
| BGIBMGA003700 | CACCTGCTGATGATATGGCAG | GCATATTGCTCCTGCTGTTC |
| BGIBMGA003701 | ATGGAGGGAGATTCCTGTCA | CCGAAACAATACTTAGGGTC |
| BGIBMGA003495 | AAGGAAGCCCTGCGAATCTA | ATAACCGTTGCCGTTTGAGT |
