# Supplementary material for: The Structural Variation Is Associated with the Embryonic Lethality of a Novel Red Egg Mutant Fuyin-lre of Silkworm, Bombyx mori
Source: PLoS One. 2015 Jun 1;10(6):e0128211. doi: 10.1371/journal.pone.0128211 (PMC4452371; doi:10.1371/journal.pone.0128211)
Supplement: S2 Table — (DOCX) [file pone.0128211.s004.docx]

S2 Table Primers used in positioning of the mutation site

| Primer name | Nscaf2674 position (bp) | Forward primer | Reverse primer |
| --- | --- | --- | --- |
| Position up1 | 5126279-5127314 | GTTCTGGAAGGCTAGGGTGT | GCACTGTTAACTGTCTTCGTC |
| Position up2 | 5127801-5128230 | GTCACCATTGTCAAGCATGTG | CCATCTTCGAGCAACTTGGCA |
| Position up3 | 5127801-5128464 | GATGCGACTAAAGCAGCAAC | CCATCTTCGAGCAACTTGGCA |
| BGIBMGA003497-1 | 5131545-5132067 | CGTCATCTGGTATGGTGGTC | CGCCATGACATGAACAAAATT |
| BGIBMGA003696 | 5235503-5235695 | GCATTTACCGTGAAAGCC | AGTATTTACAGGATGGGTTG |
| BGIBMGA003697 | 5315855-5316139 | CGTCTTGTCTCGTTACCCT | ACGTTCGCTACCATATTGAC |
| BGIBMGA003698 | 5323899-5324186 | AGCAGCAACAAGGAACCAAG | TCTGAAAACCCATAATGAACTA |
| BGIBMGA003699 | 5329846-5330200 | AAGAAGATAGTGGTGCTGAA | CAATGGTAATGTCCTTGTGTA |
| BGIBMGA003496 | 5343643-5344253 | GCACCAATAATGGCAACG | TGTGCAGCTATTTGTCTACT |
| BGIBMGA003700 | 5370537-5370809 | CACCTGCTGATGATATGGCAG | GCATATTGCTCCTGCTGTTC |
| BGIBMGA003701 | 5378186-5378357 | ATGGAGGGAGATTCCTGTCA | CCGAAACAATACTTAGGGTC |
| BGIBMGA003495 | 5381208-5381345 | AAGGAAGCCCTGCGAATCTA | ATAACCGTTGCCGTTTGAGT |
| Position down1 | 5409304-5409588 | CCGTACTGTGCAATGAATACC | GATACCCAACACCCGAACTT |
| Position down2 | 5409569-5410366 | AAGTTCGGGTGTTGGGTATC | GCTTACCCACCCATGTATGC |
| Position down3 | 5410347-5410879 | GCATACATGGGTGGGTAAGC | GAAAACGACTGGGAGATGTC |
