# Supplementary material for: The Structural Variation Is Associated with the Embryonic Lethality of a Novel Red Egg Mutant Fuyin-lre of Silkworm, Bombyx mori
Source: PLoS One. 2015 Jun 1;10(6):e0128211. doi: 10.1371/journal.pone.0128211 (PMC4452371; doi:10.1371/journal.pone.0128211)
Supplement: S3 Table — (DOCX) [file pone.0128211.s005.docx]

S3 Table DEGs with the same expression trends at 24h and 48h

| Genes | Silkworm Genome Datebase | Fuyin-*lre* / Fuyin | Up / Down |
| --- | --- | --- | --- |
|  |  | 24h / 48h Fold |  |
| BGIBMGA002222 | glutathione S-transferase theta [*Bombyx mori*] | 1.01134/1.59353 | Up |
| BGIBMGA002847 | CG31869-PA [*Drosophila melanogaster*] | 1.01748/2.49441 | Up |
| BGIBMGA005286 | RNA m5u methyltransferase [*Aedes aegypti*] | 3.18786/3.53783 | Up |
| BGIBMGA005497 | PREDICTED: similar to CG40494-PA.3 [*Apis mellifera*] | 1.46554/1.99775 | Up |
| BGIBMGA005582 | PREDICTED: similar to Suppressor of cytokine signaling 2 (SOCS-2) (Cytokine-inducible SH2 protein 2) (CIS-2) [*Tribolium castaneum*] | 1.05530/1.12031 | Up |
| BGIBMGA006105 | PREDICTED: similar to CG17652-PA [*Tribolium castaneum*] | 1.27359/1.55854 | Up |
| BGIBMGA009066 | PREDICTED: similar to CG15786-PA [*Apis mellifera*] | 1.91920/4.82783 | Up |
| BGIBMGA010168 | putative paralytic peptide-binding protein [*Bombyx mori*] | 3.68794/11.3341 | Up |
| BGIBMGA010212 | serine protease inhibitor serpin [*Bombyx mori*] | 1.94819/1.10826 | Up |
| BGIBMGA010681 | acyl-CoA desaturase [*Bombyx mori*] | 2.38041/4.80426 | Up |
| BGIBMGA011115 | TPA_inf: caterpillar 16.2 [*Mus musculus*] | 2.83994/3.12958 | Up |
| BGIBMGA012807 | PREDICTED: similar to CG4122-PG, isoform G [*Tribolium castaneum*] | 2.55043/1.95846 | Up |
| BGIBMGA012961 | myosin light polypeptide 9 isoform 2 [*Bombyx mori*] | 1.24645/2.18371 | Up |
| BGIBMGA012995 | PREDICTED: similar to CG6428-PA [*Apis mellifera*] | 2.61755/6.63804 | Up |
| BGIBMGA013037 | PREDICTED: similar to Eukaryotic translation initiation factor 4E transporter (eIF4E transporter) (4E-T) (Eukaryotic translation initiation factor 4E nuclear import factor 1) [*Tribolium castaneum*] | 1.19128/1.48026 | Up |
| BGIBMGA013276 | carboxypeptidase [*Helicoverpa armigera*] | 4.42490/5.26523 | Up |
| BGIBMGA013725 | PREDICTED: hypothetical protein [*Apis mellifera*] | 1.87807/1.96641 | Up |
| BGIBMGA001657 | PiggyBac transposable element derived 4 [*Homo sapiens*] | -3.24945/-3.72983 | Down |
| BGIBMGA001818 | n ENSANGP00000001289 [*Anopheles gambiae* str. PEST] | -1.720579/-1.28155 | Down |
| BGIBMGA002333 | Peptidase, cysteine peptidase active site; Zinc finger, CCHC-type; Peptidase aspartic, catalytic; Polynucleotidyl transferase, Ribonuclease H fold [*Medicago truncatula*] | -1.9431682/-2.9743 | Down |
| BGIBMGA003341 | PREDICTED: similar to zinc finger protein 420 [Rattus norvegicus] | -1.08298/-1.02017 | Down |
| BGIBMGA003495 | PREDICTED: protein naked cuticle homolog [*Bombyx mori*] | -7.3424551/-13.366 | Down |
| BGIBMGA003496 | PREDICTED: GPI ethanolamine phosphate transferase 1-like [*Bombyx mori*] | -11.09393/-11.9206 | Down |
| BGIBMGA003497-1 | major facilitator superfamily domain-containing protein 12-like (Bm-*re*) [*Bombyx mori*] | -11.43 / -- | Down |
| BGIBMGA003697 | PREDICTED: semaphorin-1A-like [*Bombyx mori*] | -8.022698/-7.92103 | Down |
| BGIBMGA003698 | PREDICTED: UPF0585 protein CG18661-like, transcript variant X2 [*Bombyx mori*] | -2.82 / -11.25 | Down |
| BGIBMGA003699 | PREDICTED: LIN1-like protein-like [*Bombyx mori*] | -13.39869/-13.301 | Down |
| BGIBMGA003700 | PREDICTED: ras GTPase-activating-like protein IQGAP1-like [*Bombyx mori*] | -12.17507/-12.6072 | Down |
| BGIBMGA003701 | PREDICTED: metallophosphoesterase 1-like [*Bombyx mori*] | -11.46144/-11.4486 | Down |
| BGIBMGA004282 | hypothetical protein [*Bombyx mori*] | -1.83414/-1.90998 | Down |
| BGIBMGA005052 | ecdysteroid-phosphate phosphatase [*Bombyx mori*] | -1.3135/-1.18789 | Down |
| BGIBMGA005244 | hypothetical protein KGM_06794 [*Danaus plexippus*] | -11.84152/-3.37772 | Down |
| BGIBMGA005299 | hypothetical protein 33 [*Lonomia obliqua*] | -1.76183/-1.55068 | Down |
| BGIBMGA005500 | PREDICTED: similar to CG15117-PA, isoform A [*Tribolium castaneum*] | -2.15891/-2.49378 | Down |
| BGIBMGA005506 | PREDICTED: similar to leukotriene B4 12-hydroxydehydrogenase [*Apis mellifera*] | -1.40554/-2.3421 | Down |
| BGIBMGA005511 | PREDICTED: similar to CG17280-PA [*Tribolium castaneum*] | -1.96742/-2.51493 | Down |
| BGIBMGA007794 | PREDICTED: similar to CG3182-PA, isoform A [*Tribolium castaneum*] | -10.55592/-10.7036 | Down |
| BGIBMGA008711 | putative protease inhibitor 4 [*Lonomia obliqua*] | -3.47838/-2.87815 | Down |
| BGIBMGA009033 | 3-dehydroecdysone 3alpha-reductase [*Spodoptera littoralis*] | -1.83249/-1.97234 | Down |
| BGIBMGA009139 | PREDICTED: similar to CG8947-PA [*Tribolium castaneum*] | -1.55238/-2.23201 | Down |
| BGIBMGA009956 | U2 small nuclear ribonucleoprotein A' [*Bombyx mori*] | -1.73295/-2.68313 | Down |
| BGIBMGA009988 | PREDICTED: similar to CG4049-PA [*Apis mellifera*] | -4.81291/-4.20339 | Down |
| BGIBMGA010214 | serine protease inhibitor, serpin [*Aedes aegypti*] | -2.81541/-1.90281 | Down |
| BGIBMGA012113 | expressed protein [*Oryza sativa* (japonica cultivar-group)] | -4.15724/-3.90769 | Down |
| BGIBMGA012803 | ENSANGP00000029294 [*Anopheles gambiae* str. PEST] | -2.19771/-1.98364 | Down |
| BGIBMGA012827 | fructose 1,6-bisphosphate aldolase [*Antheraea yamamai*] | -2.40182/-2.67467 | Down |
| BGIBMGA013114 | abhydrolase domain containing 11 [*Bombyx mori*] | -1.76765/-1.87102 | Down |
| BGIBMGA013816 | conserved hypothetical protein [*Aedes aegypti*] | -2.60485/-2.3139 | Down |
